# Clinical Practice Guidelines for ^18^F-Fluciclovine 2024 in the Japanese Society of Nuclear Medicine

**DOI:** 10.1007/s12149-025-02089-6

**Published:** 2025-07-25

**Authors:** Kimiteru Ito, Seishi Jinnouchi, Kaoru Kikukawa, Chio Okuyama, Yoshifumi Sugawara, Masami Kawamoto, Koichi Koyama, Kanae Kawai Miyake, Koji Murakami

**Affiliations:** 1 Department of Diagnostic Radiology, National Cancer Center Hospital, 5-1-1 Tsukiji, Chuo-Ku, Tokyo, 104-0045 Japan; 2 Department of Radiology, Atsuchi Memorial Clinic PET Center, 12-1 Terukuni-Cho, Kagoshima, 892-0841 Japan; 3 Departments of Radiology, Fujita Health University School of Medicine, 1-98 Dengakugakubo, Kutsukake-Cho, Toyoake, Aichi 470-1192 Japan; 4 Clinical Research Center, Shiga General Hospital, 5-4-30, Moriyama-Cho, Moriyama, Shiga 524-8524 Japan; 5 Department of Diagnostic Radiology, National Hospital Organization, Shikoku Cancer Center, Minamiumemotomachi Kou 160, Matsuyama, Ehime 791-0280 Japan; 6 Advanced Medical Center, Shonan Kamakura General Hospital, 13701, Okamoto, Kamakura, Kanagawa 247-8533 Japan; 7 PET Center, Osaka Saiseikai Nakatsu Hospital, 2-10-39 Shibata, Kita-Ku, Osaka, 530-0012 Japan; 8 Department of Diagnostic Imaging and Nuclear Medicine, Graduate School of Medicine, Kyoto University, 54 Kawahara-Cho, Shogoin, Sakyo-Ku, Kyoto, 606-8507 Japan; 9 Department of Radiology, Juntendo University Hospital, 3-1-3, Hongo, Bunkyo-Ku, Tokyo 113-8421 Japan

**Keywords:** ^18^F-Fluciclovine, FACBC, Axumin™, Glioma, Surgical margin

## Abstract

^18^F-Fluciclovine was the first ^18^F-labeled amino acid PET tracer to be approved for clinical use in Japan, receiving regulatory approval in March 2021 and being listed for reimbursement in June 2024. In response to this development, the Japanese Society of Nuclear Medicine initiated the formulation of clinical guidelines to ensure the appropriate use of this radiopharmaceutical in clinical practice. This guideline provides a comprehensive overview of the clinical characteristics of ^18^F-Fluciclovine in malignant glioma, including indications for use, imaging protocols, interpretation of PET images, and considerations for radiation safety. The Japanese version of this guideline was compiled by a voluntary editorial committee and officially approved by the Japanese Society of Nuclear Medicine on August 16, 2024. The primary objective of this guideline is to consolidate the current scientific evidence on ^18^F-Fluciclovine and to clarify its clinical utility, appropriate usage, and imaging methodologies. By doing so, it aims to promote the proper implementation of ^18^F-Fluciclovine in clinical settings and to serve as a reference for future applications related to the expansion of insurance coverage and reimbursement decisions.

It is recommended that PET examinations using ^18^F-Fluciclovine in Japan be conducted in accordance with this guideline. Although the content is tailored to the Japanese medical system and regulatory framework, the imaging protocols, radiation safety management, and interpretation methods described herein are also expected to be internationally applicable and relevant.

## Introduction

^18^F-Fluciclovine is a pharmaceutical agent for positron emission tomography (PET) imaging in men with suspected prostate cancer recurrence already marketed in region such as the United States and the European Union under the generic name ^18^F-Fluciclovine (brand name Axumin™). In Japan, it received regulatory approval in March 2021 as the first ^18^F-labeled amino acid PET agent, marketed under the name Axumin™, and was covered by National Health Insurance as of June 2024.

In response to this, the Japanese Society of Nuclear Medicine (JSNM) decided to formulate clinical guidelines. The purpose is to consolidate current literature regarding ^18^F-Fluciclovine and clarify its evidence-based usefulness, appropriate usage, and imaging methods. These guidelines aim not only to establish appropriate usage and indications for ^18^F-Fluciclovine but also to serve as supporting material for the National Health Insurance application by demonstrating its clinical utility.

Currently, the approved indication is “visualization of tumors in patients suspected of having newly diagnosed malignant glioma.” However, considering the potential future applications, such as “assessment of treatment response” and "early diagnosis of recurrence," the guidelines also address reports related to these uses as of now.

As the use of this agent becomes more widespread and evidence accumulates, we expect further expansion of its indications, with revisions to these guidelines anticipated in the future.

In preparing these guidelines, we were fortunate to have Dr. Seishi Jinnouchi of Atsugi Memorial Clinic and Dr. Kimiteru Ito of the National Cancer Center Hospital join as expert members, both of whom were involved in the domestic clinical trials. We would like to express our sincere gratitude to them here.

## Overview of the radiopharmaceutical

### Chemical composition

#### Content of active ingredient

This drug is an aqueous injectable formulation, sealed in a vial placed inside a radiation-shielded container. Each 2 ml vial contains ^18^F-Fluciclovine at a radioactivity of 185 MBq at the time of calibration. Excipients include 3.64 mg (5%) of Japanese Pharmacopoeia anhydrous D-mannitol, saline, and a pH adjuster per 2 ml.

#### Physical characteristics of the formulation

The solution is a clear, colorless liquid, with a pH of 2.8–4.8 and an osmotic pressure ratio of approximately 1.4 (relative to normal saline).

#### Physicochemical properties of the active ingredient

Generic name: ^18^F-Fluciclovine.

Chemical name: trans-1-amino-3-[18F]fluorocyclobutane-1-carboxylic acid.

Molecular formula: C_5_H_8_^18^FNO_2_.

Molecular weight: 132.12.

**Fig. 1:**
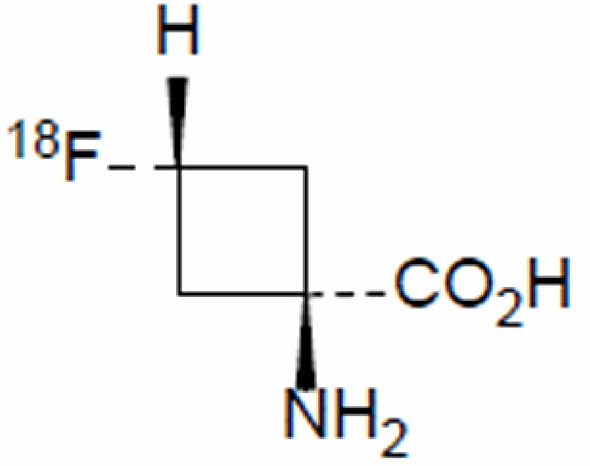
The chemical formula of ^18^F-Fluciclovine

Structural formula: **(**Fig. 1).

Nuclear Physical Characteristics (as 18F):

Physical half-life: 109.739 min.

Principal γ-ray energy: 511 keV (emission rate: 193.4%).

### Pharmacokinetics and mechanism of uptake

#### Pharmacokinetics

##### Blood concentration

When a single intravenous dose of 174.4–201.4 MBq of ^18^F-Fluciclovine was administered to six healthy adults, the mean percentages of radioactivity distribution in whole blood and plasma were 7.68%ID and 6.71%ID, respectively, at 2 min post-injection. These values gradually decreased over time, reaching 3.13%ID and 1.98%ID, respectively, at 240 min post-injection.

##### Distribution

In healthy adults, the mean radioactivity distribution per organ/tissue was highest in muscle at all imaging time points, followed by relatively high values in the liver, red bone marrow, and lungs. Among these organs and tissues, distribution to the liver, red bone marrow, and lungs peaked early after administration (at 3–10 min post-injection), with maximum mean values of 18.1%ID, 6.28%ID, and 5.56%ID, respectively, then gradually decreased.

In contrast, radioactivity in the muscle gradually increased up to 72 min post-injection (109%ID) and then slowly decreased, maintaining a high level (95.8%ID) up to 220 min post-injection. Distribution to the brain gradually increased until 220 min post-injection but remained relatively constant across all evaluation time points (ranging from 0.533%ID to 1.20%ID).

##### Absorbed dose

The absorbed doses calculated by the MIRD method are as follows. The effective dose was 0.0138 mSv/MBq. (Table 1).

**Table 1:** The absorbed doses calculated by the MIRD method

Organ	Absorbed dose (mGy/MBq)
Heart Wall	0.0239
Liver	0.0406
Muscle	0.0248
Pancreas	0.0308
Spleen	0.0213

##### Metabolism

^18^F-Fluciclovine is not metabolized in the liver and is present predominantly in its unchanged form in both plasma and urine.

##### Excretion

The cumulative urinary excretion rate of radioactivity increased over time, and the mean urinary excretion rate from immediately after administration to 24 h post-injection was 5.40%ID.

#### Mechanism of Uptake

^18^F-Fluciclovine crosses the blood–brain barrier and is taken up into cells via amino acid transporters. Since tumor cells exhibit enhanced amino acid metabolism compared to normal cells, ^18^F-Fluciclovine accumulates more in tumor tissues than in normal tissues.

### Indications

Visualization of tumors in patients suspected of having newly diagnosed malignant glioma.

It is specifically intended to assist in determining the extent of tumor resection during surgical planning based on magnetic resonance imaging (MRI) findings.

### Dosage and administration

1. Typically, one vial of this product (87–270 MBq) is administered intravenously, and PET imaging should begin 10 to 50 min after injection.
2. Precautions Related to Dosage and Administration.

Imaging should be performed within 60 min post-administration.

The imaging time should be determined considering factors such as the administered dose, imaging equipment, data acquisition conditions, and image reconstruction algorithms and parameters.

(3) Since areas showing high signal intensity on FLAIR or T2-weighted MRI images, even when confirmed as tumor by pathological examination, may not always show uptake of this agent, the possibility of false-negative results should be taken into account when planning the extent of tumor resection the extent of tumor resection based on PET imaging with this agent.

### General precautions, toxicity, and side effects

#### Important general precautions

Administer only when the diagnostic benefit is judged to outweigh the potential risks of radiation exposure.

#### Side effects

As side effects shown below may occur, careful monitoring is required. If any abnormalities are observed, administration should be discontinued and appropriate measures should be taken. (Table 2).

**Table 2:** Side Effects of ^18^F-Fluciclovine

Frequency	1% to less than 5%	Frequency unknown
Gastrointestinal	Dry mouth	
Others		Olfactory disorder, injection site erythema, injection site pain, taste abnormality

### Precautions for Patients with Specific Backgrounds

#### Pregnant Women

Administer only when the diagnostic benefit is judged to outweigh the potential risks of radiation exposure for pregnant women or women who may be pregnant.

#### Breastfeeding women

Although no specific evidence is available, it is recommended that breastfeeding be discontinued on the day of the examination, considering potential radiation exposure.

#### Pediatrics

No clinical studies have been conducted in pediatric populations.

#### Elderly patients

Administer with caution, closely monitoring the patient's condition, as physiological functions are generally diminished in the elderly.

## Guidelines on facility standards, imaging equipment maintenance, and safety management

### Standards for staffing and facilities

Since ^18^F-Fluciclovine is a radiopharmaceutical labeled with ^18^F, the facility standards for performing PET examinations should follow those established for ^18^F-FDG PET examinations, given the similar handling requirements. Under the facility standards for reimbursement of ^18^F-FDG PET examinations, the requirements include: ① At least one full-time physician with more than three years of experience in nuclear medicine diagnosis, and who has completed the required training. ② At least one dedicated radiologic technologist per imaging device who possesses specialized knowledge and experience in handling PET radiopharmaceuticals. JSNM has established certification systems for “Certified Physicians in PET Nuclear Medicine” and “Specialists in Nuclear Medicine” to train clinicians who excel in PET nuclear medicine and are proficient in PET examination safety management. Obtaining one of these certifications is recommended. Both certifications require renewal every five years, and to facilitate ongoing education, PET training seminars are regularly held and should be actively utilized.

### Image interpretation

It is desirable that images be interpreted and reports prepared by nuclear medicine specialists certified by the Japanese Society of Nuclear Medicine, such as Certified Physicians in PET Nuclear Medicine or Nuclear Medicine Specialists. For the CT component of PET/CT scans, collaboration with radiologists certified by the Japan Radiological Society, such as Certified Radiologists or Diagnostic Radiologists, is recommended when necessary. Issuing documents describing the results of PET or PET/CT examinations should ideally be done under the supervision of a physician who has experience in interpreting over 200 cases of PET or PET/CT examinations [1].

### General facility standards

Facility registration, staff qualifications, and operational methods should comply with the provisions of the Enforcement Regulations of the Medical Care Act and related notifications [2, 3].

### Maintenance and management of imaging equipment

The maintenance and management of imaging equipment should be conducted in accordance with the “Guidelines for Imaging Techniques in FDG-PET Examinations [4].”

### Safety management in PET examinations

Safety management for PET examinations should follow the "Guidelines for Ensuring Safety in FDG-PET Examinations (2005) [5]."

## Guidelines for examination procedures [6–11]

### Patient management (Pre-Examination)

Fasting prior to the administration of this agent is not stipulated in the package insert. However, considering that the patients are brain tumor patients and to prevent events such as vomiting during the examination, fasting before the scan is recommended as appropriate. In the Phase III clinical trial, fasting for more than 4 h was implemented. Vigorous exercise should be avoided before administration.

### Administration method (Injected Radioactivity Dose), precautions at administration

Administer 87–270 MBq intravenously via an upper limb vein. This corresponds to the total radioactivity of one vial (2 mL, 185 MBq/2 mL at the calibration time) between one hour before and two hours after the calibration time. After administration, flush the line with saline. There are no data regarding the efficacy or safety of this examination in pediatric patients.

### Patient management (During Waiting Time)

Since imaging starts 10 min or more after administration, a waiting period of at least 10 min is required. Considering that some patients may have unstable conditions, it is desirable to have a resting room or a designated waiting area available.

### Imaging mthod (Imaging Protocol, Positioning, etc.)

#### Positioning

The patient should be positioned supine so that the entire brain fits within the imaging field of view of the PET camera. As body movement during imaging degrades image quality, it must be minimized. Therefore, positioning should be done in a way that allows the head and neck to relax, enabling the patient to remain as still as possible during the scan.

#### Imaging protocol

Start PET/CT (or PET/MRI) imaging at least 10 min after administration and complete imaging within 60 min. The scan duration should be around 10 min. However, since PET image quality depends on camera performance and scan time, the actual imaging time should be determined considering the administered dose, scanner performance, data acquisition conditions, image reconstruction algorithms, and parameters. CT (or MRI) for positioning and attenuation correction should be performed before PET emission imaging. If there is any suspicion of patient movement during emission imaging, and it is deemed necessary, attenuation correction CT (or MRI) should be repeated after the emission scan.

### Patient management (Post-Imaging)

After imaging, a physician or a person under a physician’s direction should confirm whether there are any abnormalities in the patient. Although the patient can leave immediately after the examination, if the patient's condition permits, it is preferable to have them remain within the controlled area for about 30 min to 1 h to minimize radiation exposure to others.

### Image processing (Attenuation Correction, Reconstruction Methods, Filters, Gray Scale, etc.)

Use a matrix size of either 128 × 128 or 256 × 256 for the transverse images as standard, and perform attenuation correction.

## Image interpretation [7, 9]

Since this diagnostic agent is a novel drug, there is limited data on image evaluation, and there is room for improvement in image evaluation methods and interpretation. This guideline describes the evaluation method for which consensus was obtained in clinical trials.

### Image display method

Table 3 shows the display standards for the image color scale. Typically, ^18^F-Fluciclovine PET images are used as an aid in determining the resection extent for brain tumors. Therefore, radiologists responsible for imaging and evaluation must provide images that are easy for neurosurgeons to interpret during surgery. Although each facility may use its familiar colormap, be aware that even small differences in uptake may appear exaggerated depending on the colormap. Therefore, if necessary, verify the uptake contrast using grayscale images as well. In assessing the extent of ^18^F-Fluciclovine uptake, the colormap should be changed depending on whether the tumor shows high or low uptake. This adjustment is based on the experienced reader’s visual judgment. As a general guide, if the tumor’s SUVmax is high, a high-uptake colormap is used; if low, a low-uptake colormap is used. However, if doubts arise regarding SUV values due to imaging conditions or visual evaluation, changes to the colormap based on the reader's or surgeon’s discretion are acceptable.

**Table 3:** Image Scale Display Method for Determining the Accumulation Range

Item	Content		Display measurement method
Color map			Color Scale / Grayscale
Color scale (for high uptake)	Upper reference	50% of the SUVmax of the tumor region	Measurement of SUVmean in Normal Regions: ・Identify the center of the tumor region in the sagittal view ・Display the corresponding axial view ・Place a 10 mm diameter VOI (Volume of Interest) in the contralateral side relative to the tumor Measurement of SUVmax in Tumor Regions: ・Set a spherical VOI encompassing the entire tumor ・Measure the SUVmax ・If multiple tumors are present, select the lesion with the highest SUVmax Notes: ・Exclude physiological uptake areas such as blood vessels (including internal pooling) and the pituitary gland from the VOI ・When applying the high uptake scale, ensure that physiological uptake in the scalp or brain parenchyma does not appear anatomically abnormal compared to MRI images ・Confirm that there is no “halation” effect (overexposure) over the entire brain
	Lower reference	Twice the SUVmean of normal regions	
Color scale (for low uptake)	Upper reference	75% of the SUVmax of the tumor region (If tumor uptake cannot be clearly distinguished from normal brain parenchyma, set to 1.0)	
	Lower reference	SUVmean of normal regions	

The objective image evaluation procedure is as follows: First, measure the tumor SUVmax and the normal tissue SUVmean. Set a VOI (Volume of Interest) encompassing the entire tumor and measure the tumor SUVmax. Then, using sagittal MRI images (contrast-enhanced T1-weighted or FLAIR/T2-weighted images), identify the center of the tumor and display the corresponding axial slice. On this slice, place a 10 mm VOI in the normal region on the contralateral side and measure the SUVmean. Select normal regions based on visual assessment referencing MRI, avoiding physiologically uptake sites such as blood pools and the pituitary gland.

Next, determine the appropriate colormap: For high-uptake scales, set the upper limit to 50% of the tumor’s SUVmax, and the lower limit to twice the SUVmean of the normal region. For low-uptake scales: set the upper limit to 75% of the tumor’s SUVmax, and the lower limit to 1.5 times the SUVmean of the normal region. If it is difficult to distinguish the tumor from normal brain parenchyma, set the SUVmax of 1.0 as the scale upper limit. Adjust the lower limit to clearly depict the tumor contour (edge) based on the MRI reference if needed.

Note: In high-uptake scales, halo artifacts from scalp or brain tissue accumulation may impair evaluation of superficial brain lesions. In such cases, even if the tumor SUVmax or T/N ratio is high, a low-accumulation scale may be used for evaluation.

Standardizing display methods in this way is expected to reduce inter-reader variability.

### Image interpretation method

^18^F-Fluciclovine PET images are acquired using hybrid devices like PET/CT or PET/MRI scanners. Interpretation should be performed using PET analysis software capable of displaying fusion images with navigation MRI to anatomically localize accumulation areas based on MRI.

In brain tumors, ^18^F-Fluciclovine uptake usually corresponds to or exceeds the range of enhancing lesions on contrast-enhanced T1-weighted images (T1WI). It generally corresponds to or is narrower than the high-signal areas on FLAIR/T2-weighted images (T2WI).^18^F-Fluciclovine may detect lesions not visible on contrast-enhanced T1WI, with a positive predictive value of 88% (22 out of 25 cases). Physiological uptake is low in normal brain parenchyma but may be observed in areas like the cavernous sinus, brain surface blood pools, pituitary gland, bone marrow, and skin. Careful interpretation is required when tumors are near these structures. MRI should be referenced to verify anatomical structures during reading. Neurosurgeons create fusion images of MRI and PET using neuro-navigation systems for surgical planning. Therefore, radiologists handling ^18^F-Fluciclovine PET images should be familiar with MRI interpretation and neuro-navigation systems. For neuro-navigation, Alignment should be performed using contrast-enhanced T1WI as a reference along with ^18^F-Fluciclovine PET images, navigation T1WI, and navigation FLAIR/T2WI.

Note: Even in high-signal regions on FLAIR or T2WI confirmed as tumor on pathology, ^18^F-Fluciclovine uptake may not be observed. Hence, the possibility of false negatives must be considered when determining the extent of tumor resection based on PET imaging. If a facility planning surgery using ^18^F-Fluciclovine does not have its own PET/CT (or PET/MRI) scanner, close collaboration with the PET-equipped facility is necessary to ensure there is no doubt in image interpretation.

## Indications for examination

### Insurance-covered conditions

^18^F-Fluciclovine is indicated for the "visualization of tumors in patients suspected of having newly diagnosed malignant gliomas, specifically to assist in determining the extent of tumor resection during surgical planning based on MRI." It is therefore used for preoperative diagnosis.

Gliomas refer to primary brain tumors arising from glial cells, which support neurons, and account for approximately 25–30% of primary brain tumors in Japan [12–14]. They include various types such as astrocytomas, oligodendrogliomas, glioblastomas, ependymomas, and choroid plexus papillomas, with a wide range of malignancy grades. In the WHO 2007 classification, gliomas are graded into four categories based on pathological malignancy: Grade I (pilocytic astrocytoma), Grade II (astrocytoma, oligodendroglioma, etc.), Grade III (anaplastic astrocytoma, anaplastic oligodendroglioma, etc.), and Grade IV (glioblastoma) [15]. Surgical resection is the first-line treatment for gliomas, and maximal safe resection is considered critical for improving survival [16]. However, excessive resection may lead to neurological deficits, so careful determination of the resection area is necessary.

Note that the WHO classification was updated in 2016 to incorporate molecular diagnostics using genetic information [17], and in 2021 was further revised to emphasize genetic profiling, reorganizing into Grades 1–4 [18]. However, this guideline is based on a review of the literature using the WHO 2007 classification.

MRI, with its excellent tissue resolution, is an indispensable modality for brain tumor evaluation. Gadolinium (Gd)-enhanced T1WI plays an important role in diagnosing gliomas. However, the enhancement on MRI primarily reflects breakdown of the blood–brain barrier (BBB) rather than tumor tissue itself. Moreover, high-grade gliomas that infiltrate beyond the contrast-enhanced area and low-grade gliomas without enhancement are known to exist [19–21]. Although FLAIR imaging shows broader abnormal signals beyond the enhancing area, these may include edema as well as tumor tissue.

Given these limitations of MRI in evaluating tumor extent, there has been growing interest in the role of molecular imaging with PET. The utility of ^18^F-FDG and the amino acid tracer ^11^C-methionine (^11^C-MET) PET for glioma evaluation has been reported [22]. However, FDG PET suffers from high physiological uptake in normal brain tissue, making tumor delineation difficult. Meanwhile, ^11^C-MET PET is limited by the short physical half-life of ^11^C (about 20 min), restricting its use to facilities with an on-site cyclotron.

^18^F-Fluciclovine is a synthetic amino acid tracer labeled with ^18^F (half-life approximately 110 min), developed by Shoup et al. in 1999. In rat studies, they confirmed high accumulation in brain tumor tissue, and subsequently reported its use in a first-in-human study for detecting residual glioblastoma tissue post-surgery [23]. Following its FDA approval for evaluating prostate cancer, incidental findings of oligodendrogliomas and meningiomas have been reported during its use [24–26].

Tsuyuguchi et al. compared ^18^F-fluciclovine and ^11^C-MET PET in six preoperative glioma cases [8], and Michaud et al. evaluated 27 recurrent gliomas [27]. Both studies demonstrated that ^18^F-Fluciclovine had comparable lesion detection ability to ^11^C-MET, and showed superior tumor-to-background contrast, indicating higher tumor-to-background ratios (T/B ratios, also referred to as lesion/background [L/B] or tumor/normal tissue [T/N] ratios), facilitating better delineation of tumor margins.

In Japan, clinical trials evaluating the extent of gliomas preoperatively have been conducted. In a phase IIa clinical trial involving 37 samples from 5 untreated glioma patients, comparisons were made between MRI findings, ^18^F-Fluciclovine accumulation, and histopathology after resection [17]. All cases were positive on both Gd-enhanced MRI and PET imaging. Tissue sampling from Gd-enhancing areas, PET-positive areas, their margins, and surrounding regions revealed that the PET-positive areas were broader than the Gd-enhancing regions. Furthermore, the T/B ratios were significantly higher for tumor tissue, suggesting the potential of ^18^F-Fluciclovine PET/CT in visualizing glioma infiltration beyond what can be seen on contrast-enhanced MRI.

In a phase IIb clinical trial involving 40 preoperative glioma patients, comparisons of MRI and PET findings with resected tissues were conducted to assess tumor margins [7]. Among 26 samples that were Gd-negative but PET-positive, tumor tissue was successfully identified, demonstrating the utility of ^18^F-Fluciclovine PET in detecting tumor infiltration not visualized on contrast-enhanced MRI.

Furthermore, Wakabayashi and colleagues conducted a comparative study using data from two phase III clinical trials involving 45 cases, investigating the utility of MRI and ^18^F-Fluciclovine PET in determining the extent of resection for gliomas. They compared imaging findings with histological results and reported that the positive predictive values (PPV) for Gd( +)PET( +) and Gd(-)PET( +) areas were 100.0% (3/3 cases) and 88.0% (22/25 cases), respectively, with an overall PPV of 89.3% (25/28 cases) for PET-positive regions [9]. In this study, the intended extent of resection was modified after PET imaging in 47.2% (17/36) of the cases—expanded in 30.6% (11 cases) and reduced in 16.7% (6 cases)—demonstrating the usefulness of ^18^ F-Fluciclovine in surgical planning. Among multiple tissue samples from the same case, when histological grades differed, the higher-grade lesions generally showed greater tracer uptake.

Regarding the relationship between glioma grade and radiotracer uptake, an investigation using a dedicated brain PET scanner in 17 cases (18 specimens) ranging from grade II to IV showed that higher-grade gliomas had higher ^18^F-Fluciclovine uptake, and the degree of uptake correlated well with the Ki-67 index. It was reported that a T/B ratio of 2.15 or an SUVmax of 4.3 could serve as thresholds to distinguish between low-grade and high-grade gliomas, which can be difficult to differentiate by MRI alone [28]. Karlberg and colleagues also compared PET/MRI uptake patterns with MRI findings in grade II–IV gliomas, demonstrating that ^18^F-Fluciclovine uptake was higher in high-grade tumors and was associated with the Ki-67 index and cell density. In their study, although Gd enhancement was typically observed in higher-grade lesions, the tumor volumes evaluated by PET were 1.5 to 2.8 times larger than those assessed by Gd-enhanced MRI. PET/MRI was found to be more accurate and useful for determining resection margins compared to PET or MRI alone [29]. In cases of anaplastic oligodendroglioma, while MRI signal abnormalities indicated the tumor extent, PET-positive areas corresponded to high-grade regions within the tumor, suggesting that PET/MRI could be applied to guide biopsies and treatments targeting more malignant regions [30]. Regions with high FLAIR signals but negative PET findings often represented edema or gliosis. Tumor extent tended to be underestimated by Gd-T1WI and overestimated by FLAIR, yet in low-grade gliomas, tumor infiltration could still occur in areas negative on both Gd-enhanced MRI and PET, requiring careful interpretation [9]. Thus, it is considered that combining multiple MRI sequences with ^18^F-Fluciclovine PET in preoperative evaluation, while taking glioma grade into account, contributes significantly to the accurate determination of resection extent.

### Non-health-insurance-covered indications (Non-Reimbursed Diseases)

Although ^18^F-Fluciclovine has not been approved for the diagnosis of recurrence or evaluation of therapeutic efficacy in gliomas and is not covered by insurance, current reports are summarized below.

#### Diagnosis of recurrence

Treatment of brain tumors typically involves a combination of surgery, radiation therapy, and chemotherapy. However, postoperative changes and radiation necrosis following radiotherapy or chemoradiotherapy often present with contrast enhancement on MRI, making it difficult to distinguish these from true tumor recurrence.

In a report by Michaud et al., among 27 cases of recurrent primary brain tumors after multimodal treatment including surgery, contrast-enhanced MRI could not clearly identify recurrence in three cases, whereas ^18^F-Fluciclovine PET demonstrated abnormal uptake in all cases (SUVmax, mean 4.5 ± 2.3 [range: 1.5–10.5]; lesion-to-background ratio, mean 10.8 ± 4.5 [range: 3.6–23.8]) [27]. Of the 27 cases, 13 underwent histopathological verification of recurrence; among them, one of the two cases diagnosed as low-grade glioma showed low uptake, whereas all 11 cases diagnosed as glioblastoma, high-grade glioma, or high-grade astrocytoma exhibited clear abnormal uptake. Bogsrud et al. also reported that in 21 cases suspected of recurrent high-grade glioma after initial standard therapy, ^18^F-Fluciclovine PET successfully depicted recurrent tumors as areas of abnormal uptake (SUVmax, mean 8.3 ± 5.3; lesion-to-background ratio, median 21.6 [range: 3.1–84.4]) [31]. They also detected abnormal uptake in all four cases of small satellite lesions that were not enhanced on MRI. Henderson et al. performed MRI-guided biopsies using fused ^18^F-Fluciclovine PET/CT images in two cases of recurrent glioblastoma after multimodal therapy, finding that areas of ^18^F-Fluciclovine uptake correlated with the histopathological tumor cell density at multiple biopsy sites [32]. They suggested that ^18^F-Fluciclovine PET may help in the resection of recurrent tumors even when treatment-related changes obscure findings on MRI alone. Although not in primary brain tumors, Parent et al. conducted ^18^F-Fluciclovine PET in eight patients who underwent stereotactic radiosurgery for metastatic brain tumors originating from the lung, kidney, breast, or colon [33]. They reported a diagnostic accuracy of 100% (cutoff SUVmax 1.3 at 30 min post-injection) for distinguishing true tumor recurrence from radiation necrosis. From these findings, ^18^F-Fluciclovine PET appears potentially useful as an adjunct for the diagnosis of recurrence, especially in high-grade tumors like glioblastoma and high-grade gliomas, although further evidence accumulation is awaited.

Regarding comparisons with ^11^C-MET PET, in Michaud et al.'s study of 27 cases of recurrent primary brain tumors, data were available for 16 cases where both ^18^F-Fluciclovine PET and ^11^C-MET PET were performed on the same day [27]. ^18^F-Fluciclovine PET showed high uptake in 25 cases and low uptake in 2 cases, whereas ^11^C-MET PET demonstrated high uptake in 10 cases but low uptake in 6 cases. Although the SUVmax for recurrent lesions was similar between ^18^F-Fluciclovine (4.5 ± 2.3) and ^11^C-MET (5.0 ± 2.2), the background SUVmean was lower with ^18^F-Fluciclovine (0.5 ± 0.2) compared to ^11^C-MET (1.3 ± 0.4), resulting in better lesion-to-background contrast for ^18^F-Fluciclovine. These results suggest that ^18^F-fluciclovine PET may offer easier detection of recurrent lesions compared to ^11^C-MET PET.

#### Evaluation of therapeutic response

Pseudoprogression refers to benign changes characterized by progressive enlargement of enhancement areas on MRI within 12 weeks after completing radiotherapy, which subsequently improve without treatment. Because both pseudoprogression and true tumor progression show contrast enhancement on MRI, differentiating between them is often challenging. Recent small-scale studies have reported that ^18^F-Fluciclovine PET can accurately differentiate pseudoprogression from true tumor progression; however, these reports involve only a few cases, and further research is needed [34]. Currently, no extensive reports regarding the use of ^18^F-Fluciclovine PET for general therapeutic response evaluation have been identified in the literature.

## Data Availability

Not applicable.
